# Prevalence of Antidepressant Use during Pregnancy in Denmark, a Nation-Wide Cohort Study

**DOI:** 10.1371/journal.pone.0063034

**Published:** 2013-04-25

**Authors:** Espen Jimenez-Solem, Jon Trærup Andersen, Morten Petersen, Kasper Broedbaek, Nadia Lyhne Andersen, Christian Torp-Pedersen, Henrik Enghusen Poulsen

**Affiliations:** 1 Laboratory of Clinical Pharmacology Q7642, Rigshospitalet, Copenhagen, Denmark; 2 Department of Clinical Pharmacology, Bispebjerg Hospital, Copenhagen, Denmark; 3 Department of Clinical Biochemistry, Rigshospitalet, Copenhagen, Denmark; 4 Mental Health Center Copenhagen, Copenhagen University Hospital, Copenhagen, Denmark; 5 Department of Cardiology, Gentofte Hospital, Copenhagen, Denmark; 6 Faculty of Health Sciences, University of Copenhagen, Copenhagen, Denmark; University of Manitoba, Canada

## Abstract

**Aim:**

The aim of this study was to assess the prevalence and patterns of exposure to antidepressants before, during and after pregnancy in a cohort including all pregnant women in Denmark between 1997 and 2010.

**Methods:**

We performed a retrospective cohort study including 912 322 pregnancies. Information was retrieved from the Danish Birth Registry and The Register of Medicinal Product Statistics to identify women redeeming an antidepressant prescription during pregnancy. Exposure periods were based on standard treatment doses and dispensed pack sizes.

**Results:**

We identified 19 740 pregnancies exposed to an antidepressant at some point during pregnancy. The rate of exposure increased from 0.2% in 1997 to 3.2% in 2010. We found that the rate of exposure was halved during the first 3 months of pregnancy. In contrast, we describe a clear increase in exposure after pregnancy among pre-delivery treatment-naïve women.

**Conclusions:**

In spite of uncertainty concerning antidepressants’ safety during pregnancy we find a 16-fold increase in exposure rates between 1997 and 2010. The rates describe a sharp decrease in exposure during pregnancy that is probably caused by physicians’ hesitation to prescribe antidepressants and women’s fear of unwanted effects on the unborn child. More studies are needed to clarify the consequences of antidepressant discontinuation during pregnancy.

## Introduction

It is estimated that approximately 20% of women of childbearing age (25–45 years), [1], [2] and up to 15% of pregnant women suffer from depressive symptoms [3], [4]. Untreated depression can have serious consequences for the mother, the newly born and their family. Depression during pregnancy is associated with preterm delivery, low birth weight, epidural analgesia, caesarean section, intensive ward admission, and disturbances in the child’s neurocognitive and socioemotional development [4]–[8]. Untreated depression during pregnancy is associated with a 6-fold risk increase of postpartum depression [4], [9].

Research dealing with the consumption of antidepressants and subsequent pregnancy outcomes has indicated an increased risk of congenital malformations, and more notably heart defects [10]–[22]. However, the results are conflicting [12], [14], [17]–[19], [23]–[33] and studies including up to a million pregnancies indicate little risk of congenital malformations [12], [17], [19], [31], [32], or the possibility of confounding by indication [34]. On the other hand, studies show a clear association between SSRI use and persistent pulmonary hypertension of the newborn [35], and no association with perinatal mortality [36], [37].

It is important to know the prevalence of prenatal exposure to antidepressants in order to estimate its potential public health consequences. Therefore, we set out to quantify the percentage of pregnant women in treatment with antidepressants in Denmark. We assessed temporal trends over the years 1997–2010, and use in relation to pregnancy. Additionally, we looked at maternal characteristics associated with antidepressant exposure.

## Materials and Methods

We identified all pregnant women exposed to an antidepressant in Denmark between 1997 and 2010 using the Danish Medical Birth Registry and the Register of Medicinal Product Statistics.

### Study Population

The study population comprised all women giving birth in Denmark between 1^st^ January, 1997 and 1^st^ January, 2010 (n = 920 639). All citizens in Denmark have a unique personal civil registration number that enables individual-level linkage of information across nationwide registries [38]. Linkage between three of these registries was used in the present study. All births between 1997 and 2010 were identified using the Danish Medical Birth Registry, which contains a unique identification number for the mother, father and child, age and prior births as well as birth length, death and cause of death, sex and gestational age of the offspring [39]. Time of conception is based on ultrasound estimates or information on the date of the last menstrual period. Data on all hospitalizations was retrieved from The Danish National Hospital Register [40]. The registry includes admission and discharge dates, and hospitalization discharge diagnoses coded according to the International Classification of Diseases, 10th revision (ICD-10). The Register of Medicinal Product Statistics was used to draw information on every prescription dispensed from Danish pharmacies since 1997 [41]. Registered information includes type of drug, strength, quantity dispensed, and dispense dates. The international Anatomical Therapeutic Chemical (ATC) classification system was used to code all antidepressants. Danish pharmacies are required to register all dispensed prescriptions as part of the national healthcare reimbursement scheme for drug expenses. This ensures high rates of registration [42].

Subjects were divided into quartiles according to their annual household gross income during the year of birth. Education was divided into three groups according to the highest obtained level of education.

Information on smoking and body mass index (BMI) was obtained from the Danish Medical Birth Registry Smoking was divided into five classes according to the number of daily cigarettes: no smoking, 1–10, 11–20, and >20. Information on BMI was only available from 1^st^ January 2004, and divided into 4 classes according to kg/m^2^: <18.5, 18.5–24.9, 25.0–29.9, and >29.9.

### Identification of Antidepressant Pharmacotherapy

Patients being treated with antidepressants were included if they claimed one of the antidepressants listed in table 1. The following antidepressants were not included in our study due to low exposure rates (n<10): nefazodone (N06AX06), ruboxetine (N06AX18), duloxetine (N06AX21), moclobemid (N06AG02), doxepin (N06AA12). Bupropion (N06AX12) was not included since its indication in Denmark is smoking cessation. We allowed for exposure to multiple antidepressants.

**Table 1:** Antidepressants included in the study.

Antidepressant	ATC code
**SSRIs**	**N06AB**
Citalopram	N06AB04
Escitalopram	N06AB10
Fluvoxamine	N06AB08
Fluoxetine	N06AB03
Paroxetine	N06AB05
Sertraline	N06AB06
**TCAs**	**N06AA**
Amitriptylin	N06AA09
Clomipramin	N06AA04
Dosulepin	N06AA16
Imipramin	N06AA02
Nortriptylin	N06AA09
**Other**	**N06AX**
Mianserin	N06AX03
Mirtazapine	N06AX11
Venlafaxine	N06AX16

Table shows the generic names and their corresponding ATC codes for antidepressants included in the present study. SSRI: Selective Serotonine Reuptake Inhibitor; TCA: Tri Cyclic Antidepressant (Non-selective Monoamine Reuptake Inhibitor).

To estimate exposure prevalence we calculated dosages for each individual in the cohort, based on dispense date of each prescription, strength and number of tablets prescribed. For each prescription, we calculated an exposure period based on the average dosage of up to seven previous consecutive prescriptions. If the prescription was the first in a series, we calculated exposure time by using the standard daily dose for the drug. The standard daily dose was selected for each available medication strength in accordance with the accepted standard daily therapeutic dose [43]. The number of dispensed pills was divided by the daily dosage to calculate treatment periods. We assumed a treatment period to be continuous, for a series of prescriptions, if this was compatible with at least the minimal dose of the prescribed tablets (i.e. one daily tablet). We defined treatment breaks shorter than 60 days as being in treatment. This was done to take subjects with lower daily dosages than anticipated into account. We chose 60 days *á priori*, because it seemed a reasonable amount of days to account for subjects decreasing dosage for a period of time or using stored medication. To ensure that the *a priori* period of 60 days was reasonable, we estimated exposure prevalence with treatment breaks of 30 or 90 days as being in treatment. The estimates did not differ. We assumed treatment periods to continue during hospitalization. We have previously used this method to calculate treatment periods during pregnancy [34], [36].

We defined exposure to antidepressants during a given period as presence of one or more days of treatment. If treatment extended over two or more periods (i.e. two trimesters), exposure was considered to have occurred in all affected periods.

### Ethics

Registries were linked and personal data analysed on computers held by Statistics Denmark, where data was made available with encrypted personal information [43]. This ensured that no individuals could be identified. In Denmark The Act on Processing of Personal Data does not require ethical permission or obtained written informed consent for anonymised retrospective registry studies. The present study has been approved by The Danish Data Protection Agency (No. 2008-41-2517).

### Statistical Analysis

All analyses and data management were performed using SAS 9.2 (SAS Institute Inc., Cary, NC, USA).

We used frequencies and percentage to present baseline characteristics. To assess differences in baseline characteristics for categorical variables we used chi-square tests. Statistical significance was defined as *p*<0.05. All statistical tests were two sided.

## Results

### Material Overview

We identified 912 322 pregnancies resulting in a birth between 1997 and 2010, of which 19 740 were exposed to an antidepressant at some point during pregnancy. Table 2 shows the number and basic characteristics of pregnant women treated with antidepressants divided into 3 categories according to exposure at any time during pregnancy: SSRI (n = 16 928), TCA (n = 1 297) and other antidepressants (n = 3 135).

**Table 2:** Basic characteristics for women exposed to an antidepressant during pregnancy.

	SSRI	TCA	Others	Any AD	No exposure	
	(N = 16 928)	(N = 1297)	(N = 3135)	(N = 19740)	(N = 892582)	
Characteristic	n (%)	n (%)	n (%)	n (%)	n (%)	p-value
**Education**						<0.001
Unskilled	7645 (45.16)	557 (42.95)	1572 (50.14)	8991 (45.55)	291155 (32.62)	
Skilled	4524 (26.72)	393 (30.30)	818 (26.09)	5287 (26.78)	265794 (29.78)	
Higher	4345 (25.67)	312 (24.06)	647 (20.64)	4950 (25.08)	295149 (33.07)	
Missing values	414 (2.45)	35 (2.70)	98 (3.13)	512 (2.59)	40484 (4.54)	
**Annual household income**						<0.001
Less than $ 62 192	6174 (36.47)	402 (30.99)	1315 (41.95)	7248 (36.72)	216452 (24.25)	
$ 62 192–$ 89 140	4313 (25.48)	405 (31.23)	836 (26.67)	5081 (25.74)	218620 (24.49)	
$ 89 141–$ 126 344	3290 (19.44)	244 (18.81)	510 (16.27)	3768 (19.09)	219935 (24.64)	
$ 126 345 or greater	2816 (16.64)	225 (17.35)	391 (12.47)	3215 (16.29)	220490 (24.70)	
Missing values	335 (1.98)	21 (1.62)	92 (2.93)	428 (2.17)	1785 (0.20)	
**Age (years)**						<0.001
<20	530 (3.13)	15 (1.16)	97 (3.09)	599 (3.03)	24447 (2.74)	
21–25	2748 (16.23)	163 (12.57)	538 (17.16)	3185 (16.13)	135330 (15.16)	
26–30	5486 (32.41)	374 (28.84)	995 (31.74)	6338 (32.11)	335476 (37.58)	
31–35	5396 (31.88)	441 (34.00)	963 (30.72)	6308 (31.96)	286027 (32.04)	
>35	2768 (16.35)	304 (23.44)	542 (17.29)	3310 (16.77)	111302 (12.47)	
Missing values	0 (−)	0 (−)	0 (−)	0 (−)	0 (−)	
**Parity**						<0.001
1	7430 (43.89)	497 (38.32)	1397 (44.56)	8646 (43.8)	389794 (43.67)	
2	5483 (32.39)	386 (29.76)	860 (27.43)	6255 (31.69)	327381 (36.68)	
>2	3858 (22.79)	402 (30.99)	849 (27.08)	4658 (23.6)	169875 (19.03)	
Missing values	157 (0.93)	12 (0.93)	29 (0.93)	181 (0.92)	5532 (0.62)	
**Daily cigarettes**						<0.001
0	11147 (65.85)	859 (66.23)	1869 (59.62)	12890 (65.3)	705637 (79.06)	
1–10	4245 (25.08)	315 (24.29)	937 (29.89)	5043 (25.55)	130280 (14.60)	
11–20	294 (1.74)	19 (1.46)	71 (2.26)	350 (1.77)	5240 (0.59)	
>20	680 (4.02)	47 (3.62)	138 (4.40)	793 (4.02)	22269 (2.49)	
Missing values	562 (3.33)	57 (4.39)	120 (3.83)	664 (3.37)	29156 (3.27)	
**BMI (kg/m2)**						<0.001
<18.5	811 (4.79)	34 (2.62)	154 (4.91)	932 (4.72)	27743 (3.11)	
18.5–24.9	6279 (37.09)	361 (27.83)	1156 (36.87)	7202 (36.48)	247064 (27.68)	
25.0–29.9	2678 (15.82)	187 (14.42)	574 (18.31)	3123 (15.82)	85611 (9.59)	
30	2078 (12.28)	153 (11.80)	435 (13.88)	2414 (12.23)	47632 (5.34)	
Missing values	5082 (30.02)	562 (43.33)	816 (26.03)	6069 (30.74)	484532 (54.28)	

Table shows the number (n) of pregnant women within each subcategory. Some women were exposed to several antidepressants during pregnancy. Number in parenthesis denotes percentage of pregnant women within each exposure. Information on BMI was only available for women giving birth after 1 January 2004. Chi-square tests were used to assess the overall p value for the group comparison between unexposed and women exposed to “any AD”.BMI, body mass index; SSRI, selective serotonin reuptake inhibitor; TCA, tricyclic antidepressants; AD, antidepressant.

### Maternal Characteristics

Pregnant women treated with antidepressants were characterized by being older, having more prior pregnancies, having lower annual household income, having a shorter educational career, smoking more and having a higher BMI than unexposed women (table 2).

### Exposure Rates, 1997–2010

**Figure 1:**
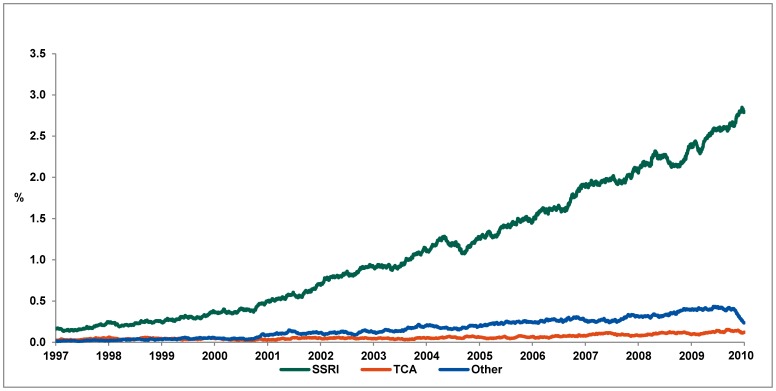
Point prevalence of pregnant women in treatment with an antidepressant based on estimated treatment periods. SSRI, selective serotonin reuptake inhibitor; TCA, tricyclic antidepressants; AD, antidepressant.

Between January 1997 and January 2010 the percentage of pregnant women exposed to an antidepressant increased from 0.2% in early 1997 to 3.2% in December 2009 (figure 1). This 16-fold increase was sustained by the increase in exposure to SSRIs that accounted for 88.7% of exposure among pregnant women in December 2009. TCAs and other antidepressants accounted for 3.8% and 7.5% respectively.

For the specific SSRIs we see a change in choice of treatment between 1997 and 2009. In 1997, Citalopram (48.6%) and Fluoxetine (31.9%) were the preferred SSRIs during pregnancy, followed by Sertraline (9.7%) and Paroxetine (9.7%). In 2002 34.0% of pregnancies were exposed to fluoxetine, 26.9% to citalopram, 20.3% to sertraline, 18.5% to paroxetine and 0.3% to escitalopram. In December 2009 the prevalence changed; fluoxetine (15.7%), citalopram (47.4%), sertraline (26.8%), paroxetine (1.8%) and escitalopram (8.2%).

The prevalence of exposure to TCAs remained steady since 1997 at an average of 0.03% of pregnancies. Amitriptyline and nortriptyline represented over 70% of TCA exposure.

Exposure to other antidepressants increased in the study period reaching a maximum peak point prevalence of 0.4% of pregnant women in August 2009. We observed an abrupt fall in prevalence at the end of 2009. The most common exposure among other antidepressants was venlafaxine, with 63.0% of total exposure in the study period, followed by mirtazapine (19.8%) and mianserin (7.0%).

### Exposure in Relation to Pregnancy

**Figure 2:**
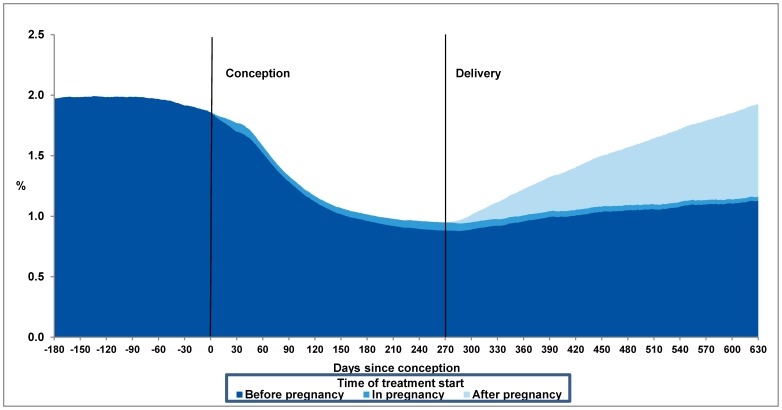
Percentage of pregnant women in treatment with an antidepressant for each day from 180 days before conception to 630 days after conception (approximately 1 year after mean time of delivery). The figure is divided into three areas of different color indicating the period of treatment start; before (dark blue), during (blue) or after pregnancy (light blue).

At the time of conception, 16 962 (1.86%) of all pregnancies were exposed to an antidepressant, of these, 51% were still exposed at the time of delivery. The greatest decrease in antidepressant exposure is consistent with the period of pregnancy recognition (1–3 months after conception) (figure 2). Table 3 shows the number of exposed women during each trimester. 1694 (0.19%) pre-pregnancy treatment naïve women commenced treatment with an antidepressant at some point during pregnancy. Within the first six months after delivery 5 053 (0.55%) commenced treatment among women who had never been in treatment with an antidepressant before the time of delivery. Within 12 months the number had risen to 11 151 (1.22%) (table 4). In contrast to women exposed to antidepressants during pregnancy, these women were younger than unexposed women (p<0.001). They have however, a lower annual household income, a shorter educational career, smoke more and have a higher BMI than unexposed women (p<0.001). Figure 2 shows exposure prevalence to any antidepressant before, during and after pregnancy for all pregnancies in the cohort.

**Table 3:** Number of women exposed to an antidepressant during pregnancy.

	Trimester		
	First	Second	Third
Any antidepressant	18273	13039	9721
SSRI	15403 (84.29)	11370 (87.20)	8641 (88.89)
Citalopram	6657 (36.43)	4306 (33.02)	2850 (29.32)
Escitalopram	1539 (8.42)	722 (5.54)	344 (3.54)
Fluoxetine	3898 (21.33)	3618 (27.75)	2927 (30.11)
Paroxetine	1779 (9.74)	1164 (8.93)	816 (8.39)
Sertraline	3059 (16.74)	2565 (19.67)	2328 (23.95)
TCA	1101 (6.03)	748 (5.74)	479 (4.93)
Amitriptyline	578 (3.16)	292 (2.24)	120 (1.23)
Clomipramin	125 (0.68)	89 (0.68)	57 (0.59)
Dosulepin	40 (0.22)	42 (0.32)	36 (0.37)
Imipramin	61 (0.33)	32 (0.25)	15 (0.15)
Nortriptyiline	327 (1.79)	308 (2.36)	254 (2.61)
Other	3039 (16.63)	1655 (12.69)	934 (9.61)
Mianserin	270 (1.48)	113 (0.87)	57 (0.59)
Mirtazapine	876 (4.79)	348 (2.67)	142 (1.46)
Venlafaxine	1687 (9.23)	1109 (8.51)	703 (7.23)

Number in parenthesis is the percentage of ‘any antidepressant’. Sum of percentages adds up to more than 100% due to some pregnancies being exposed to more than one antidepressant in the given period. SSRI, selective serotonin reuptake inhibitor; TCA, tricyclic antidepressants; AD, antidepressant.

**Table 4:** Pre-pregnancy treatment-naïve women exposed to an antidepressant.

	Start during pregnancy	Start after pregnancy	
		0–6 months	0–12 months
Any antidepressant	1694	5053	11151
SSRI	1416 (83.59)	4246 (0.47)	9304 (1.02)
Citalopram	489 (28.87)	1936 (0.21)	4588 (0.50)
Escitalopram	78 (4.60)	495 (0.05)	1235 (0.14)
Fluoxetine	499 (29.46)	367 (0.04)	825 (0.09)
Paroxetine	107 (6.32)	487 (0.05)	1083 (0.12)
Sertraline	363 (21.43)	1130 (0.12)	2167 (0.24)
TCA	155 (9.15)	265 (0.03)	704 (0.08)
Amitriptyline	71 (4.19)	136 (0.01)	426 (0.05)
Clomipramin	9 (0.53)	18 (0.00)	48 (0.01)
Dosulepin	10 (0.59)	10 (0.00)	25 (0.00)
Imipramin	20 (1.18)	22 (0.00)	62 (0.01)
Nortriptyiline	48 (2.83)	93 (0.01)	188 (0.02)
Other	183 (10.80)	901 (0.10)	2295 (0.25)
Mianserin	28 (1.65)	136 (0.01)	348 (0.04)
Mirtazapine	108 (6.38)	513 (0.06)	1191 (0.13)
Venlafaxine	45 (2.66)	263 (0.03)	800 (0.09)

Number of pre-pregnancy treatment-naive women exposed to an antidepressant during and after pregnancy. Number in parenthesis is the percentage of ‘any antidepressant’. Sum of percentages adds up to more than 100% due to some pregnancies being exposed to more than one antidepressant in the given period. SSRI, selective serotonin reuptake inhibitor; TCA, tricyclic antidepressants; AD, antidepressant.

**Figure 3:**
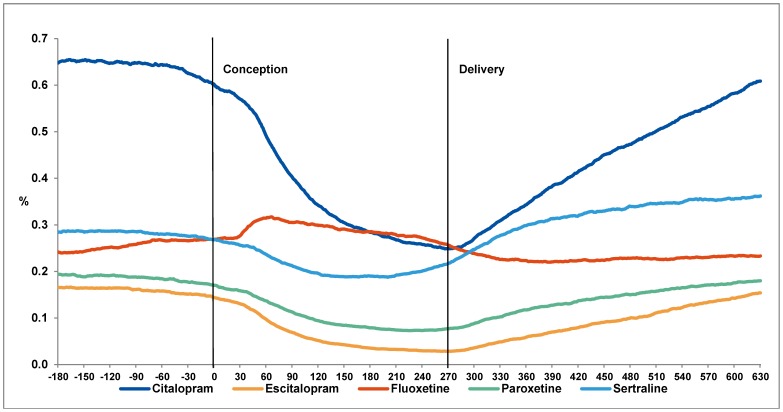
Percentage of pregnant women in treatment with an SSRI for each day from 180 days before conception to 630 days after conception (approximately 1 year after mean time of delivery). Lines depict the different types of SSRIs.

This pattern of exposure from 6 months before to 12 months after pregnancy was similar for the individual antidepressants, except for fluoxetine (figure 3). For fluoxetine, we saw a rise in prevalence after conception followed by a slight decrease until delivery.

#### Exposure to more than one antidepressant

We identified 3388 (0.37%) pregnancies exposed to more than one antidepressant during pregnancy; 2995 to two, 364 to three and 29 to four different antidepressants.

We identified 1 629 (0.18%) pregnancies exposed to two SSRIs at some time during pregnancy. The most frequently used combinations during pregnancy were fluoxetine and citalopram (n = 663), and fluoxetine and sertraline (n = 554). We identified 62 pregnancies exposed to mirtazapine and venlafaxine among pregnancies exposed to other antidepressants, but no pregnancies exposed to two TCA’s during pregnancy.

#### Antidepressant switch during pregnancy

While 43.3% (n = 8552) stopped antidepressant treatment during pregnancy, 11.3% (n = 2224) of pregnant women switched to a different antidepressant; 6.8% during the first trimester and 4.5% during the second trimester. Among those who switched treatment, the preferred new antidepressant was fluoxetine (41.7%) followed by citalopram (20.5%) and sertraline (19.1%).

Few pregnant women switched antidepressant from fluoxetine (6%), sertraline (6%) or citalopram (8%), compared to mianserin (42%), escitalopram (25%) or mirtazapine (26%).

## Discussion

We have described patterns of exposure to specific antidepressant among all pregnant women in Denmark between 1997 and 2010. We found an increase in prevalence of exposure during the study period from 0.2% to 3.2%. Furthermore we described a decrease in exposure to antidepressants related to the time of pregnancy recognition, and a steep increase in exposure among pre-pregnancy treatment-naïve after delivery.

### Exposure Rates, 1997–2010

The increase in prevalence over the years is comparable to studies from other countries, although our estimates are considerably lower [44]–[49]. Two studies from the USA describe prevalences of 7.6% in 2005 [45] and 13.3% in 2003 [47]. Two studies from The Netherlands reported prevalences of 2% between 2000 and 2003c and 3% in 2004 [46]. The first three studies were based on health insurance records and the fourth on data from a Dutch region. One study from the UK estimated a prevalence of 3.3% in 2006, based on data from general practices [49]. None of the mentioned studies were based on nation-wide cohorts as the present study. Differences in prevalence could be accounted for by study methodology, and socio-demographic differences.

The increasing exposure to antidepressant during pregnancy until 2010 was mainly due to redemption of SSRIs, where citalopram was the most frequently used SSRI in 2009. Use of paroxetine has stagnated since 2004, and accounted for only 5.3% of SSRI use in 2008 (figure 2), and could be due to reports published in 2005 by the FDA associating paroxetine with heart defects [50]. Use of TCAs and other antidepressants increased at a more moderate rate between 1997 and 2010.

One possible reason for this increase over the last 13 years is the widening of the therapeutic indications for antidepressants to include anxiety disorders, premenstrual syndrome, posttraumatic stress disorders, migraine prophylaxis, pain and eating disorders [51]. We hypothesize that a second reason could be a more liberal prescription of antidepressants during pregnancy. In spite of many studies reporting increased risks of congenital malformations associated with antidepressants, the absolute risk increases are low. On the other hand, in spite of doctor recommendation, only 35% of pregnant women reported to be willing to take antidepressants during pregnancy in an American study [52]. Thirdly, it has been suggested that influence by the pharmaceutical industry could play a role in the increased use of antidepressants during pregnancy [53].

Increased rates of exposure to some newer antidepressants (e.g. escitalopram) will open for the possibility of safety studies on these drugs and their possible association with less frequent pregnancy outcomes (e.g. specific congenital malformations and persistent pulmonary hypertension of the newborn).

### Use in Relation to Pregnancy

Overall, at the time of pregnancy recognition we see a considerable decrease in prevalence of SSRI exposure, and an increase after delivery.

Approximately half of all pregnancies discontinued treatment during pregnancy, which is in accordance with previously published literature [46], [49], [54]–[57]. In our study, this decrease was not found for fluoxetine, for which the prevalence increased. This could indicate a switch in treatment to fluoxetine when pregnancy is detected, which is in accordance with recommendations from The Danish Society of Obstetrics and Gynecology (DSOG). DSOG recommends the use of fluoxetine or sertraline during pregnancy [58] which could explain why exposure to sertraline decreased only 26.5% during pregnancy in contrast to citalopram (60.7%), escitalopram (81.4%) and paroxetine (56.0%). During the first year after pregnancy (period of lactation) we see the steepest increase in use for citalopram (figure 3), which is not in accordance with DSOGs guidelines for treatment during lactation. During lactation DSOG recommends the use sertraline or paroxetine [58].

During pregnancy only 1694 (0.19%) treatment-naïve women commenced use of an antidepressant. This could indicate physicians and women’s reluctance towards starting treatment during pregnancy, unless symptoms are severe. It is of note that most of these women continued treatment at least one year after delivery (figure 2).

Pregnancy does not seem to protect against the risk of depression relapse [59]. Discontinuation of antidepressant treatment during pregnancy is associated with a 5-fold increase in the risk of relapse of depression during pregnancy compared to women who maintained their medication [60] and is estimated to create a substantial economic burden on society due to added use of the health care system by mother and child [61]. The cause of discontinuation could be fear of harm to the fetus or physicians’ recommendations [62]. In our study, 27% of women discontinuing treatment during pregnancy resumed treatment within one year after delivery. This could indicate a low rate of relapse in our Danish cohort comprising antidepressant users without information on depression severity. On the other hand, only 1.25% of treatment-naïve women commenced antidepressant treatment during the first year after delivery.

### Strengths and Limitations

We had no information on compliance or the women’s intention of commencing a treatment shortly after redemption of an antidepressant. This could lead to an overestimation of treatment periods. However, a Dutch study estimated that the majority of pregnant women redeeming a prescription take their medication [63]. A small Danish study estimated that compliance in Denmark is 80% for antidepressant treatment during pregnancy [64]. We did not have information on women discontinuing their treatment to commence treatment with herbal medications against depression (e.g. St John’s Wort). Importantly, information on drug use for women experiencing a spontaneous or provoked abortion was not available in our databases, and exposure rates during pregnancy could differ from those reported in our study.

The main strength of our study is the completeness of the registries, including nearly all births in Denmark and the mothers’ drug redemptions in the study period. 97.5% of all redeemed prescriptions are included in The Register of Medicinal Product Statistics [42]. All prescriptions recorded in this registry have been redeemed and paid for, which increases the probability of exposure. All redeemed prescriptions in Denmark are, by law, required to be registered as part of the national healthcare reimbursement scheme. Information is recorded prospectively and not based on questionnaires or interviews, which eliminates recall bias. Data gathered from the registries include information on drug-names and quantities redeemed. This information is difficult to obtain through questionnaires or interviews of women who have to remember use of medications over a long period of time [65]–[68].

Although the overall prevalence of antidepressant use through the last 13 years has increased, half of all pregnant women discontinue their use during pregnancy, probably due to uncertainty of the safety of antidepressants. Based on these findings it seems important for women of childbearing age and physicians to have information of high standards to help them in treatment decisions during pregnancy.
